# New Appliances and Things Medical

**Published:** 1895-08-03

**Authors:** 


### NEW APPLIANCES AND THINGS MEDICAL.

[We shall be glad to receive, at our Office, 428, Strand, London, W.C., from the manufacturers, specimens of all new preparations and appliances which may be brought out from time to time.]

### VIBRONA TONIC WINE.

(Fletcher, Fletcher, and Co., Holloway, London, N.)

Port wine and bark has long been regarded as a favourite tonic, combining the astringent and stimulating properties of the wine with the tonic properties of the cinchona bark. This union has, however, many objectionable features, amongst which the unpleasant taste of the combination is perhaps the most important, and has retarded its general acceptance as a tonic wine. Messrs. Fletcher, Fletcher, and Co. have hit upon the happy idea of combining their hydrobromic extract of cinchona with a good generous port wine in such a way as to minimise the taste of the bark extract to such an extent that the wine remains in a very palatable condition. We are strongly of the opinion that such a combination, containing as it does the total extract of ten grains of bark per wineglassful, is to be preferred to other tonic wines in which the amount of alkaloid is unknown. The well-known properties of the cinchona alkaloids also warrant the belief that such a wine as Vibrona cannot have any secondary results on the system even when taken for a long period, and therefore points to its being indicated for convalescents from acute illness and for growing children. We understand the proprietors have devised a system of analysis by their own expert which ensures the wine being of uniform quality, and our own analysis of the samples sent us for examination confirms the favourable opinion we had formed from tasting and examining the sample. Our analyst is of the opinion that a full-bodied genuine port wine has been used in its manufacture, and that each wineglassful can be guaranteed to contain the whole extract from ten grains of cinchona bark.

The total extract found by him amounts to 17·89 per cent., and the alcoholic strength is equivalent to 16·14 per cent. of alcohol by weight. The acidity of the wine was also determined, and found to be similar to that of a good vintage port wine, whilst the total alkaloids present were such as should be yielded by an average cinchona bark. A considerable quantity of the cinchona tannin and acids were also present, showing that an extract of bark had been employed which retains these constituents, which by many members of the profession are believed to possess therapeutic properties which are not shared by the isolated alkaloids. We consider that the firm have certainly added a most elegant form of the old "Port Wine and Bark" to the resources of the physician, and have no doubt, having tried it clinically, that it is a most valuable form of a well-known therapeutic remedy.

### KARLSWOOD CREOSOTE.

(E. Griffiths Hughes, Chemist, Victoria and Cateaton Streets, Manchester.)

The beneficial results that may be obtained from the exhibition of creosote in various affections of the respiratory tract are well known and widely recognised. The preparation named Karlswood Creosote is a volatile fluid, which very readily gives off creosote in a gaseous form, and thus this new inhalant is especially useful in all cases where it is desired to employ creosote in this form.

